# The Odium Medicum and Homœopathy in America

**Published:** 1888-08-11

**Authors:** 


### THE ODIUM MEDICUM AND HOMŒOPATHY IN AMERICA.

An esteemed American correspondent sends us an account of a happy hospital family free from all taint of the odium medicum. We, of course, regard the whole business as bordering on the ludicrous, and are utterly opposed to the views he expresses. We merely reproduce his letter as a curious little picture of American ways and manners:—

"Doubtless some people have wondered, and perhaps regretted, the admission of homœopathic practice into the Newton Hospital, Boston; but there seemed to be no other course to pursue. It was a settled question from the start. The hospital began with twenty subscriptions of $500 each, and one-half of those subscribers, or nearly that proportion, employ homœopathic physicians in their families. So far as one can judge, Newton was pretty equally divided between the two schools of practice; and in a hospital which was to be supported by the citizens generally, the question of course decided itself. There was never a dissenting voice in the board of trustees about it, and the plan has worked admirably. There has never been a moment's friction in the working of the hospital on account of it. The physicians of the two schools make their visits, and their treatment is carried into effect with no more clashing than if they were attending different patients in the different rooms of an hotel or in different houses. One side of the pharmacy is stocked with the tinctures and pills of the old school, and the other side with the pellets and dilutions of the new; and the matron and nurses attend to their administration with an impartiality that is delightful to witness. One of the most successful surgeons at the hospital, who has recently performed ovariotomy with marked success, and with an unusually rapid recovery, is a homœopathist. The homœopathic school in Boston is doing very good work, and sending out some very intelligent and successful practitioners. For a good deal of its success on this point, the hospital is indebted to the good judgment and impartial line of conduct pursued by the matron, Miss Pray."

